# Supplementary material for: Role of Cervical Elastography in Predicting Progression to Active Phase in Labor Induction in Term Nulliparous Women
Source: Diagnostics (Basel). 2025 Feb 19;15(4):500. doi: 10.3390/diagnostics15040500 (PMC11854331; doi:10.3390/diagnostics15040500)
Supplement: Supplementary file 1 [file diagnostics-15-00500-s001.zip › diagnostics-3418140-supplementary.pdf]

Supplementary Table S1. Baseline characteristics and delivery outcomes of study population.

	Total (N=71)
**Maternal baseline characteristics**	
Maternal age (years)	33 (31–36)
Pre-gestational BMI (kg/m²)	22.1 (20.3–25.5)
BMI at delivery (kg/m²)	27.5 (25.1–31.2)
Dinoprostone use	41 (57.7%)
Bishop score	2 (1–3)
**Ultrasound variables**	
Cervical length (cm)	2.41 (1.92–3.31)
Estimated fetal weight (kg)	3.36 (3.17–3.67)
PCA (degree)	118 (102–134)
AOP (degree)	99 (91–105)
ECI	3.82 (3.10–4.51)
HR	56.0 (42.8–64.7)
IOS	0.363 (0.285–0.458)
EOS	0.303 (0.243–0.357)
**Delivery variables**	
Gestational age (days)	279 (273–282)
Vaginal delivery	44 (62.0%)
Birth weight (kg)	3.32 (3.00–3.57)
Neonatal sex (male)	34 (47.9%)

Data are presented as medians (interquartile ranges) and numbers (percentages) where applicable.

Abbreviations: AOP, angle of progression; BMI, body mass index; ECI, elasticity contrast index; HR, hardness ratio; IOS, mean strain from the internal os; EOS, mean strain from the external os; PCA, posterior cervical angle.

Supplementary Table S2. Results of the multivariate logistic regression analysis of predictors.

Model	OR (95% CI)	p-value	Delong test	AUROC	AUPRC	Sensitivity	Specificity	PPV	NPV
Bishop score (≥ 3)	8.87 (2.70–35.4)	0.001*	Reference #1	0.694	0.570	0.483	0.905	0.778	0.717
IOS/EOS ratio (≥ 1)	0.525 (0.162–1.66)	0.272	(versus Reference #1) 0.119	0.555	0.594	0.276	0.833	0.533	0.625
Bishop score (≥ 3) and IOS/EOS ratio (≥ 1)	15.5 (4.22–69.7)<br>0.198 (0.051–0.714)	< 0.001*<br>0.015	(versus Reference #1) 0.029*	0.776	0.480	0.759	0.738	0.667	0.816
Model 1: Age, BMI, gestational age	0.959 (0.842–1.09)<br>0.869 (0.749–0.984)<br>1.06 (0.984–1.16)	0.514<br>0.042*<br>0.134	Reference #2 (versus Reference #1) 0.749	0.720	0.535	0.552	0.833	0.696	0.729
Model 2: Age, BMI, gestational age), Bishop score (≥ 3)	10.3 (2.77–48.1)	0.001*	(versus Reference #2) 0.088	0.808	0.542	0.552	0.857	0.727	0.735
Model 3: Age, BMI, gestational age), Bishop score (≥ 3), IOS/EOS ratio (≥ 1)	15.3 (3.84–76.5)<br>0.256 (0.061–1.02)	< 0.001*<br>0.055	(versus Reference #2) 0.043*	0.834	0.517	0.690	0.857	0.769	0.800
